# A critical review of flipped classroom challenges in K-12 education: possible solutions and recommendations for future research

**DOI:** 10.1186/s41039-016-0044-2

**Published:** 2017-01-07

**Authors:** Chung Kwan Lo, Khe Foon Hew

**Affiliations:** University of Hong Kong, Pokfulam, Hong Kong

**Keywords:** Student Achievement, Student Engagement, Student Attitude, Instructional Approach, Operational Challenge

## Abstract

An increasing number of teachers are using flipped classroom approach in their teaching. This instructional approach combines video-based learning outside the classroom and interactive group learning activities inside the classroom. The purpose of the present review is to provide an overview of flipped classroom studies in K-12 education. Particularly, we put emphasis on revealing and addressing the potential challenges of flipped classroom approach. Fifteen journal publications of K-12 flipped classrooms were analyzed in terms of their flipped learning activities, student achievement, student attitude, and challenges encountered. The results suggested that a variety of pre-class (e.g., online exercises) and in-class (e.g., brief review, individual practices) activities were provided in addition to instructional videos and small-group activities respectively. The use of flipped classroom approach in K-12 education yielded a neutral or positive impact on student achievement when compared to traditional classroom. Mixed results of student attitude toward flipped classroom approach were discovered. Challenges of implementing flipped classrooms were identified and categorized into student-related challenges, faculty challenges, and operational challenges. Based on the suggestions of previous studies together with relevant empirical supports, we propose a rudimentary flipped classroom model and a set of 10 guidelines to address these challenges. Finally, several recommendations of future research are provided.

## Review

Flipped classroom approach has become a popular pedagogy in many education institutes around the world. The basic notion of flipped classroom approach is to deliver the teacher’s lectures before class through online videos, in order to free-up the in-class time for active learning and problem solving activities.

The use of flipped classroom approach has been extensively studied, especially in the contexts of higher education. Following the previous reviews (e.g., Bernard 2015; Betihavas et al. 2016; Bishop and Verleger 2013; Chua and Lateef 2014; Giannakos et al. 2014; O’Flaherty and Phillips 2015; Presti 2016; Seery 2015; Zainuddin and Halili 2016; Zuber 2016), we knew that flipped classroom approach enables teachers to spend more in-class time on student-centered instructions such as group discussion and teachers’ individual assistance; that student perceptions and engagement toward flipped classroom approach are generally positive; and that some indirect educational outcomes such as improving students’ communication skills, promoting more independent learners, and changing in learning habits (e.g., revisit the online learning materials before examination) can result from the application of this instructional approach.

When compared the learning outcomes with traditional teaching, the previous reviews suggest that flipped classroom approach can improve student performance or at worst do no harm to student learning. In the published research of flipped classrooms, only a few studies (e.g., Gundlach et al. 2015) reported that students in traditional classroom preformed significantly better than the students in its flipped counterpart.

The major problems of using flipped classroom approach include teachers’ considerable workload of creating flipped learning materials, and students’ disengagement in the out-of-class learning. In fact, the previous reviews report that some students did not familiarize with this new learning approach and skipped the pre-class activities. In some flipped courses, a substantial amount of pre-class preparation efforts had caused students to be dissatisfied with the flipped classroom approach.

While the previous reviews have provided some useful snapshots of flipped classroom research, these reviews appear to be inadequate to inform us about the practice of flipped classroom approach in K-12 education. Some review studies limited their search only to the higher education context (e.g., Bernard 2015; Chua and Lateef 2014; O’Flaherty and Phillips 2015; Seery 2015). Some other reviews examined subject disciplines that are usually offered in post-secondary education such as nursing (e.g., Betihavas et al. 2016; Presti 2016). So far, only two articles about K-12 flipped classrooms (i.e., Bergmann and Sams 2009; Kong 2014) were found and reviewed. At the time of writing, no literature review study has been done that focuses specifically on the flipped classrooms in K-12 education. A systematic review is thus necessary to investigate the implementation of K-12 flipped classrooms.

The present review contributes to the literature by examining (a) the flipped learning activities of K-12 flipped classrooms, (b) the effects of K-12 flipped classrooms, (c) K-12 students’ attitude toward the flipped courses, and (d) the challenges of implementing K-12 flipped classrooms. We then propose a rudimentary flipped classroom model and a set of guidelines to inform the future practices of flipped classroom approach in K-12 education.

### An overview of flipped classroom research

There is a variety of flipped classroom research. To handle the complexity of the existing studies, we first provide an overview of flipped classroom studies through the analytical lens of de Bono’s (2000) “Six thinking hats” model. This model is a systematic thinking approach comprised of six directions: Information, feelings, constructive, creative, thinking about thought, and challenges. Six different colored hats are used to represent a direction of thinking (Table 1). This systematic thinking model helps us identify the research gap of current flipped classroom research.

**Table 1:** An overview of flipped classroom research through “six thinking hats” model

Thinking hat	Descriptions	Examples and representative citations
White hat (information)	Focusing on facts and information about flipped classroom approach	Administrating a quasi-experiment to compare flipped classroom and traditional classroom (Bhagat et al. 2016); describing the types of out-of-class and in-class activities of flipped classroom approach (DeLozier and Rhodes 2016)
Red hat (feelings)	Considering students’ emotions and feelings of flipped courses	Investigating student engagement and course satisfaction of flipped courses (Gilboy et al. 2015; Gross et al. 2015)
Blue hat (thinking about thought)	Thinking about the thoughts required in flipped classroom approach	Discussing the pedagogies and theories that can be applied in flipped classroom approach (Bishop and Verleger 2013; Abeysekera and Dawson 2015)
Green hat (creative)	Integrating new elements into flipped classroom approach	Attempting to use student-created digital videos (Engin 2014) or mobile-assisted learning system (Wang 2016) in flipped courses
Black hat (challenges)	Focusing on the challenges of using flipped classroom approach	Identifying challenges of implementing flipped classrooms in nursing education, such as more lecture preparation efforts were required (Betihavas et al. 2016)
Yellow hat (constructive)	Constructing design guidelines for flipped classroom approach	Proposing design principles or guidelines for flipped classroom approach, such as providing an incentive for students to prepare for class (Kim et al. 2014)

The white hat concerns about information. A majority of studies provided explicit information of flipped classroom approach. For example, some researchers (e.g., Bhagat et al. 2016) administered quasi-experiments to reveal the efficacy of flipped classroom approach. DeLozier and Rhodes (2016) articulate different types of in-class and out-of-class learning activities found in the literature of flipped classroom approach. These studies enhance our understanding of the effects and current practices of flipped classroom approach.

The red hat is about emotions and feelings. Some flipped classroom studies focused on student engagement and satisfaction. For example, Gilboy et al. (2015) enhanced student engagement of their courses by using flipped classroom approach. Gross et al. (2015) found a high level of student engagement and course satisfaction in their flipped classroom. From these studies, we learn that some teachers were able to promote student engagement and course satisfaction by flipping their courses.

The blue hat focuses on the thoughts required to explore a particular issue. In the contexts of flipped classroom research, Bishop and Verleger (2013) discuss various pedagogies (e.g., cooperative learning, problem-based learning) which can be used to enhance the design of flipped classrooms. Also, they recommend using objective measures to evaluate the effects of flipped classroom approach. Abeysekera and Dawson (2015) propose adopting cognitive load theory and self-determination theory as a framework to design a flipped course. Prior to a large-scale implementation of flipped classrooms, they propose a research agenda which consists of three directions: (1) small-scale localized interventions, (2) larger scale meta-studies or systematic reviews, and (3) qualitative work into student learning and their experiences.

The green hat represents creative thinking which tries out new methods of implementing flipped classrooms. For example, Engin (2014) tried to develop students’ language skills through “student-created digital videos,” instead of the usual teacher-created videos. In other words, her students were not only a consumer of teacher-prepared materials but also a producer of learning resources. In Wang’s (2016) study, he attempted to use a mobile-assisted learning system in his flipped course. Students were thus able to study anytime and anywhere through the learning system.

The black hat is a symbol of critical thinking with a specific focus on difficulties and problems. In de Bono’s (2000) point of view, the attitude of the black hat is critically important because it “protects us from doing silly things” (p. 75). Among the published studies, very few review papers identify the challenges of implementing flipped classrooms based on empirical evidence across studies. In Betihavas et al.’s (2016) review, they categorized the challenges reported into three main themes: Student-related challenges, faculty challenges, and operational challenges. Although these three main themes basically covered all aspects of flipped classroom challenges, Betihavas et al. (2016) cautioned that their “review was limited by the small number of studies” (p. 20) specifically in nursing education. Nevertheless, their analysis has enabled a further research on the challenges of using this instructional approach.

Finally, the yellow hat adopts a constructive way of thinking. In their study, Kim et al. (2014) generated nine design principles for flipped classroom approach. These principles included providing an incentive for students to prepare for class, providing clearly defined and well-structured guidance, providing facilitation for building a learning community, among others. Kim et al. (2014), however, stressed that the nine principles were limited because they were built upon a single context of one urban university in the United States. What are some guidelines for the implementation of flipped classrooms in K-12 education? The present review intends to address this very question.

### Purpose of review and research questions

The flipped classroom approach is considered as an innovation in K-12 education since 2012 (Horn 2013). The purpose of the present review is to understand the use of flipped learning activities, the effects of flipped classroom approach on K-12 students’ achievement and their attitude toward this new instructional approach. In addition, the challenges of using flipped classroom approach in K-12 education were identified. Based on the voices of teachers and students together with the existing literature, the overarching goal of the present review is to propose a flipped classroom model and a set of guidelines that could address these potential challenges. The present review is guided by the following questions:

1. What are the flipped learning activities used in K-12 flipped classrooms?
2. What is the effect of flipped classroom approach on K-12 students’ achievement?
3. What is the K-12 students’ attitude toward flipped classroom approach?
4. What are the main challenges of using flipped classroom approach in K-12 education?
5. How can we design a flipped classroom and address these possible challenges?

### Methods

#### Definition of flipped classroom approach

The flipped classroom approach can be described as “events that have traditionally taken place inside the classroom now take place outside the classroom and vice versa” (Lage et al. 2000, p. 32). However, merely a re-ordering of the teaching and learning activities is insufficient to represent the practice of this instructional approach. Bishop and Verleger (2013) thus attempt to formulate a definition of flipped classroom approach. As they define, flipped classroom approach is a technology-supported pedagogy that consists of two components: (1) direct computer-based individual instruction outside the classroom through video lectures and (2) interactive group learning activities inside the classroom. In particular, their definition is rigorous in terms of the requirement of using instructional videos in the out-of-class learning component.

By adopting Bishop and Verleger’s (2013) definition, we can distinguish flipped classroom approach from some age-old strategies of class preparation. Traditionally, students were expected to prepare for class meetings by reading the textbook on their own. However, asking students to read text-based materials on their own does not involve the elements of lecturing such as teachers’ explanation and elaboration of concepts. Hence, this kind of students’ pre-class self-study cannot really capture the idea of inverting “the order in which the instructor participated in the learning process” (Jensen et al. 2015, p. 9) of flipped classroom approach. In contrast, by using instructional videos, teachers can introduce students with new knowledge and elaborate the concept with examples before class meetings. More in-class time can thus be spent on group learning activities and solving real-world application problems with the support of teacher and peers. Therefore, we regard the use of audio or video materials (e.g., instructional videos, YouTube, screencast, Khan Academy, podcast) for out-of-class learning and regular (instead of optional) face-to-face class meetings as the two necessary elements of flipped classroom approach.

#### Search strategy

The process of selecting relevant literature followed the Preferred-Reporting of Items for Systematic Reviews and Meta-Analyses statement (PRISMA) (Moher et al. 2009). In order to be as comprehensive as possible, the following eight electronic databases were searched: (1) Academic Search Complete, (2) British Education Index, (3) Business Source Complete, (4) Communication & Mass Media Complete, (5) ERIC, (6) Library, Information Science & Technology Abstracts, (7) Teacher Reference Center, and (8) TOC Premier.

The search terms used in the present review were as follows: (“flip*” OR “invert*”) AND (“class*” OR “learn*”) AND (“K12” OR “K-12” OR “primary” OR “elementary” OR “secondary” OR “high school” OR “middle school”). In this way, the common phases of expressing flipped classroom (e.g., inverted classroom, flipped learning, flipping a class) as well as K-12 education (e.g., elementary school, secondary education) could be included.

#### Study selection and inclusion criteria

The inclusion and exclusion criteria of study selection were developed (Table 2). To be included in the present review, the studies must be published in peer-reviewed journals and written in English. The time period of our search was January 1994 to September 2016 (time of writing) since the studies prior to 1994 were unlikely to reflect the flipped classroom approach (O’Flaherty and Phillips 2015). In addition, the studies must be an empirical research reporting an implementation of flipped classrooms in any contexts of K-12 education. The flipped course must satisfy Bishop and Verleger’s (2013) definition of flipped classroom approach. Therefore, we excluded the studies that utilized only text-based materials in their out-of-class learning activities or did not offer regular face-to-face lessons.

**Table 2:** Inclusion and exclusion criteria for selection

Criterion	Inclusion	Exclusion
Definition of flipped classroom	The flipped classroom should at least include (1) the use of audio or video materials for students’ class preparation, and (2) regular face-to-face class meetings.	The flipped classroom that utilized only text-based materials in out-of-class learning activities, or did not have any regular face-to-face lessons.
Participants	Students in K-12 education settings (e.g., elementary schools, secondary school, high school)	All other students outside the contexts of K-12 education (e.g., higher education, continuing education)
Time period	January 1994 to September 2016.	The studies that outside the time period.
Type of article	The studies must be empirical research published in peer-reviewed journals	The studies that were not peer reviewed
Language	English	Non-English studies

#### Search outcomes

**Fig. 1:**
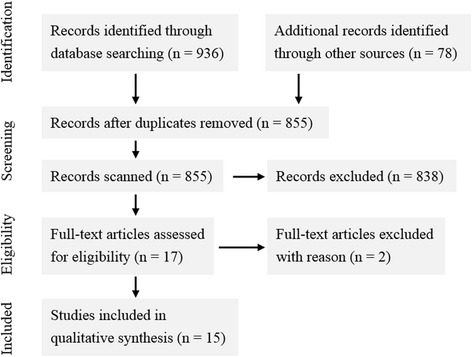
PRISMA flow diagram of article selection

By using the search terms, a total of 936 peer-reviewed journal articles were found as of October 1, 2016. However, a number of articles were removed due to replication across databases. Also, a large number of articles were found to be irrelevant after reviewing the title and abstract, particularly those were not empirical research or did not involve K-12 students. A snowballing procedure was also executed by tracking the existing literature reviews of flipped classroom research which did not limit their study within the contexts of higher education (i.e., Bishop and Verleger 2013; Giannakos et al. 2014; Zainuddin and Halili 2016). An additional 78 records were then identified and scanned. However, only two articles were found to be an empirical study of K-12 flipped classrooms. As a result, 17 full-text articles were assessed for eligibility, but two of the studies were excluded since only text-based materials were provided for students’ class preparation. The final selection yielded a total of 15 articles. Figure 1 outlines the process of article selection.

#### Data extraction and analysis

The two authors contributed to the extraction and categorization of data. Data included author(s), year of publication, research context, flipped learning activities (i.e., pre-class, in-class, and after-class activities), major findings, problems encountered, and proposed solutions or preventive strategies to the problems. In particular, the problems identified were analyzed and categorized into three themes defined by Betihavas et al. (2016): (1) student-related challenges, (2) faculty challenges, and (3) operational challenges. The data in each theme were then summarized and synthesized. In the event of disagreements regarding the data extraction and analysis, the authors re-examined the studies in question together in order to come to a consensus.

### Findings

The present review yielded 15 empirical studies of K-12 flipped classrooms. The major findings of these studies were summarized in Appendix 1. Table 3 overviews the background of these studies. A majority of the studies were conducted in the USA (*n* = 7), followed by Taiwan (*n* = 6), Canada (*n* = 1), and England (*n* = 1). With regard to the subject domain, most of the flipped courses were related to the STEM field (Science, *n* = 2; Technology, *n* = 1; Engineering, *n* = 1; Mathematics, *n* = 6). Other subjects included social studies (*n* = 2), Chinese, (*n* = 1), English (*n* = 1) and health education (*n* = 1).

**Table 3:** An overview of the reviewed studies of K-12 flipped classrooms

Study	Context	Subject	Sample size (approach)	Student age and grade level (if available)	Research design (duration)
Bhagat et al. (2016)	High school (Taiwan)	Math	41 (FC) 41 (TC)	Aged 14–15	QE (6 weeks)
Chao et al. (2015)	High school (Taiwan)	Engineering	46 (FC) 45 (TC)	Aged ~17 Grade 11	QE (8 weeks)
Chen (2016)	High school (USA)	Health	33 (FC) 31 (TC)	Grade 9	QE (3 weeks)
Clark (2015)	Secondary school (USA)	Math	42 (FC)	Aged 13–15 Grade 9	CS (7 weeks)
DeSantis et al. (2015)	High school (USA)	Math	26 (FC) 21 (TC)	Grades 9–11	QE (1 topic)
Grypp and Luebeck (2015)	High school (USA)	Math	21 (FC)	Not mentioned	AR (3 weeks)
Huang and Hong (2016)	High school (Taiwan)	English	40 (FC) 37 (TC)	Aged ~16 Grade 10	QE (12 weeks)
Kettle (2013)	High school (England)	Physics	12 (FC)	Aged 16–18 AS/A2 level	PE (appeared to be one semester)
Kirvan et al. (2015)	High school (USA)	Math	29 (FC) 25 (TC)	Grades 7–8	QE (appeared to be one topic)
Lai and Hwang (2016)	Elementary school (Taiwan)	Math	20 (SRFC) 24 (FC)	Grade 4	QE (4 weeks)
Mazur et al. (2015)	High school (Canada)	Social studies	5 classes (FC)	Grade 9	AR (1 year)
Schultz et al. (2014)	High school (USA)	Chemistry	29 (FC) 32 (TC)	Aged 15–18 Grades 10–12	QE (4 months)
Snyder et al. (2014)	High school (USA)	Social studies	209 (FC)	Grade 9	Action research (3 years)
Tsai et al. (2015)	Elementary school (Taiwan)	Computer	50 (FPBL) 48 (PBL) 46 (TC)	Grade 6	QE (15 weeks)
Wang (2016)	High school (Taiwan)	Chinese	29 (MAFC) 27 (FC)	Aged 15–16 Grade 11	QE (2 weeks)

*FC* flipped classroom, *FPBL* problem-based learning with flipped classroom, *MAFC* mobile-assisted flipped classroom, *PBL* problem-based learning, *SRFC* self-regulated flipped classroom, *TC* traditional classroom, *AR* action research, *CS* comparison study (historical control), *PE* pre-experimental (single group study), *QE* quasi-experimental design

As Table 3 shows, 13 out of 15 studies were conducted in high school or secondary school, and the other two studies were conducted in elementary school. However, not all studies reported a complete profile of their student participants in terms of the age and grade level. Based on the available information, we found that the practice of flipped classrooms usually starts from grades 9 to 12 (aged 13 to 18). Five studies (i.e., Chao et al. 2015; Huang and Hong 2016; Kettle 2013; Schultz et al. 2014; Wang 2016) involved upper secondary students (Grade 10 to 12), four studies (i.e., Chen 2016; Clark 2015; Mazur et al. 2015; Snyder et al. 2014) involved ninth graders, and one study (i.e., DeSantis et al. 2015) involved Grade 9 to 11 students. Only Kirvan et al. (2015) implemented a flipped course for Grade 7 and 8 students. In the two studies of elementary school flipped classrooms, both studies involved upper primary students – fourth graders for Lai and Hwang (2016), and sixth graders for Tsai et al. (2015). None of the studies involved lower primary students. In terms of grade level, Grade 4 is currently the lower bound of flipped classroom research.

In following sections, we organized our findings based on our research questions (i.e., the flipped learning activities, the effects on student achievement, student attitude, and the challenges of using flipped classroom approach).

#### Flipped learning activities in K-12 flipped classrooms

**Fig. 2:**
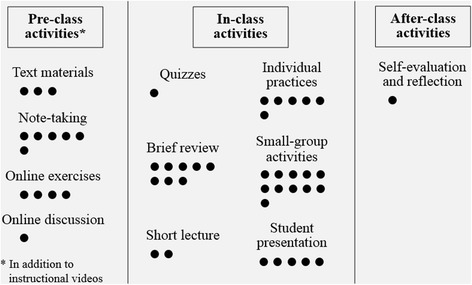
A summary of the flipped learning activities in the reviewed studies

Figure 2 presents the flipped learning activities (i.e., pre-class, in-class, and after-class) offered in the reviewed studies. In addition to watching instructional videos, we identified several types of learning activities that were commonly used in the reviewed studies. For the pre-class activities, the major activities included reading text-based materials (*n* = 3) such as textbook and notes, taking notes (*n* = 6), and doing online exercises (*n* = 4). As for the in-class activities, the main activities included brief review (*n* = 8), individual practices (*n* = 6), small-group activities (*n* = 11), and student presentation (*n* = 5). For the after-class activities, only one studies reported that students were required to do self-evaluation and reflection after finishing each unit (Lai and Hwang 2016). The detailed flipped learning activities of each study are summarized in Appendix 2.

#### Effects of flipped classroom approach on K-12 student achievement

To investigate student achievement in K-12 flipped classrooms, we focused specifically on comparison studies (e.g., quasi-experimental) that involved at least one group of flipped classroom and one group of traditional classroom. As shown in Table 3, the present review included 11 comparison studies. However, two of them (i.e., Lai and Hwang 2016; Wang 2016) compared their flipped classroom with an altered format of flipped classroom rather than a traditional classroom. In the rest of the nine studies, eight studies (i.e., Bhagat et al. 2016; Chao et al. 2015; Chen 2016; DeSantis et al. 2015; Huang and Hong 2016; Kirvan et al. 2015; Schultz et al. 2014; Tsai et al. 2015) employed a quasi-experimental design to compare student achievement in flipped classroom with its traditional counterpart, and one study (i.e., Clark 2015) compared the flipped classroom with its traditional format in previous cohort (historical control). Five studies reported that the students in flipped classroom either performed overall significantly better than the students in traditional classroom (Bhagat et al. 2016; Chao et al. 2015; Schultz et al. 2014; Tsai et al. 2015) or performed better on certain aspect (Huang and Hong 2016). Four studies found no significant difference in student achievement between the flipped classroom and traditional classroom (Chen 2016; Clark 2015; DeSantis et al. 2015; Kirvan et al. 2015). In the present review, no study reported a detrimental or inferior effect of flipped classrooms on student achievement.

However, one should exercise caution in viewing our findings. The following three limitations in some K-12 studies could have affected their comparison of student achievement. First, not all studies utilized a pre-test or pre-treatment assessment to evaluate the initial equivalence among groups (see Bhagat et al. 2016; Chao et al. 2015; DeSantis et al. 2015; Huang and Hong 2016; Kirvan et al. 2015 for exceptions). The comparability of comparison groups thus became uncertain, which hindered further analysis (e.g., meta-analysis) on student achievement (Cheung and Slavin 2013).

Second, the duration of interventions was short in general, ranging from 4 weeks to 4 months. As Clark (2015) acknowledged, a novelty effect might result in a short-term boost to student performance when new technology was instituted. Meanwhile, Tsai et al. (2015) alerted that some teachers in flipped classroom might spend more time and efforts on their experimental (i.e., flipped) groups. The neutrality of data might thus be influenced.

Third, a majority of the comparison studies in the present review were conducted in the contexts of K-12 mathematics education (e.g., Bhagat et al. 2016; Clark 2015; DeSantis et al. 2015; Kirvan et al. 2015). More empirical studies from other subject disciplines such as English are required to examine the general effects of K-12 flipped classrooms on student achievement (Huang and Hong 2016).

#### K-12 students’ attitude toward flipped classroom approach

To investigate K-12 students’ attitude toward flipped classroom approach, we examined students’ self-reported data (e.g., surveys, interviews), instructors’ reflections, and researchers’ observations reported in the reviewed studies. We found that students were generally satisfied with the use of flipped classroom approach (e.g., Bhagat et al. 2016; Schultz et al. 2014; Snyder et al. 2014; Clark 2015). More specifically, qualitative comments suggested the following three advantages of flipped classroom approach which contributed to a high satisfaction of the flipped courses.

First, students reported that watching the video lectures before class helped them prepare for the class activities (e.g., Chao et al. 2015; Grypp and Luebeck 2015; Huang and Hong 2016; Tsai et al 2015; Wang 2016) and that it was easier than reading text-based materials (Snyder et al. 2014). In particular, Schultz et al. (2014) found that “most students had a favorable perception about the flipped classroom noting the ability to pause, rewind, and review lecture” (p. 1334). These functions enabled students to take notes at their own pace (Snyder et al. 2014) and watch the instructional videos multiple times to gain a better understanding (Clark 2015).

Second, flipped classroom approach helped increase interactions with the classmates and teacher during class meetings (Chao et al. 2015; Chen 2016; Clark 2015; Schultz et al. 2014). In-class activities such as group discussion promoted students’ interactions with their peers (e.g., Clark 2015; Grypp and Luebeck 2015; Kettle 2013). In additional to the subject knowledge, students could “discuss and clarify learning goals in a collaborative manner” (Mazur et al. 2015, p. 13). In turn, these teamwork skills might promote student performance in various contexts such as extracurricular activities (Clark 2015). Besides, teacher could offer timely assistance in flipped classrooms (Tsai et al. 2015). For example, Clark’s (2015) students reported that the teacher’s individual assistance improved their understanding on the topics. Bhagat et al. (2016) further elaborated that flipped classroom approach could help the low achievers because they were able to get more attention from teachers.

Third, there were greater opportunities for students to apply the new knowledge in solving problems (Chao et al. 2015; Mazur et al. 2015; Schultz et al. 2014) and engage in the discussion of higher level problems (Tsai et al. 2015). Consistent with Kettle’s (2013) students’ opinion, Bhagat et al. (2016) pointed out that working through problems in class was an effective and enjoyable learning activity of flipped classroom approach. Clark’s (2015) students also showed their preference toward flipped classroom approach since it provided more chances for a variety of instructional practices (e.g., project-based learning, real-world applications) rather than merely listening to lectures.

Contrary to these positive findings, DeSantis et al. (2015) discovered that the satisfaction of their flipped classroom was significantly lower than that of their traditional classroom. They illustrated that students generally reacted negatively toward the change of instructional approach. Chen (2016) also reported that some students resisted initially because they did not get used to learning at home prior to the lesson. Consequently, some of them skipped the pre-class activities and came unprepared to the class. It thus resulted in a negative impact on the group dynamics of the in-class activities.

#### Challenges of using flipped classroom in K-12 education

Following Betihavas et al.’s (2016) analysis, the challenges identified in the reviewed studies were categorized into three main themes, namely student-related challenges, faculty-related challenges, and operational challenges. Each category of challenge was further coded into sub-categories.

Table 4 lists five student-related challenges in K-12 flipped classrooms. For example, some negative comments were related to video lectures: “Watching videos was considered the least effective and least enjoyable classroom activity” (Kettle 2013, p. 594), and “the video stood out as being particularly unhelpful” (DeSantis et al. 2015, p. 50). For the out-of-class supports, “students were not able to ask their questions immediately while watching the lesson videos” (Bhagat et al. 2016, p. 141).

**Table 4:** Student-related challenges in K-12 flipped classrooms

	Category	Descriptions	Supported citations
1.	Familiarity of flipped classroom	Some students held a conventional view of learning.	Snyder et al. 2014; Wang 2016
		Some students did not get used to the routines of flipped classroom approach.	Clark 2015; Schultz et al. 2014; Snyder et al. 2014
2.	Video lectures	Instructional videos were too long; and students could not focus on watching videos.	Kettle 2013; Schultz et al. 2014
		Watching videos were boring and passive.	Snyder et al. 2014
3.	In-class activities	Some students needed more clear instructions on how to work productivity in groups during class.	Grypp and Luebeck 2015
4.	Student workload	Pre-class activities were time consuming and overwhelmed students’ time at home.	Schultz et al. 2014; Snyder et al. 2014; Wang 2016
5.	Out-of-class supports	Students could not ask questions immediately during video lectures.	Bhagat et al. 2016; Schultz et al. 2014

Table 5 illustrates the two faculty challenges related to teachers’ familiarity of flipped classroom approach and their preparation of flipped classroom. In fact, most of the faculty challenges were related to teachers’ preparation of flipped classroom. For example, “it is not an easy task to find videos that perfectly match what a teacher wants his or her students to learn, and it is extremely time consuming to create their own instructional videos” (Chen 2016, p. 418) and “Each ten-minute screen-cast took hours to produce. Most of this production was done at home because long stretches of undisturbed time was needed” (Snyder et al. 2014, p. 314).

**Table 5:** Faculty challenges in K-12 flipped classrooms

	Category	Challenges	Supported citations
1.	Familiarity of flipped classroom	Teachers might not understand the value of flipped classroom and accustomed to this new instructional approach.	Grypp and Luebeck 2015
2.	Preparation of flipped classroom	Limited materials (e.g., instructional videos, handouts) were available and suitable for a particular class.	Chen 2016; Grypp and Luebeck 2015
		Preparing flipped learning materials required considerable start-up effort.	Chen 2016; Kettle 2013; Kirvan et al. 2015; Snyder et al. 2014

Table 6 summarizes the four operational challenges identified in K-12 flipped classrooms. For example, several studies revealed problems about students’ IT resources: “it was found that although most participants had their own mobile devices, many did not have enough Internet access authorization at home” (Wang 2016, p. 411), and “students being unable to load and play the videos at home if they had any kind of technological problems” (Chen 2016, p. 418).

**Table 6:** Operational challenges in K-12 flipped classrooms

	Category	Challenges	Supported citations
1.	Students’ IT resources	Student might not have Internet access to view the videos at home.	Chen 2016; Clark 2015; Kettle 2013; Snyder et al. 2014; Wang 2016
2.	Monitoring students outside class	It was difficult to ensure that students had truly watched the video.	Chao et al. 2015
3.	Teachers’ IT skills	Teacher might not be able to upload the videos online.	Chen 2016
4.	Institutional supports	Flipped classroom approach relied on the extent of the investment by schools in computer resources.	Huang and Hong 2016

### Discussion

In the present review, we investigated the flipped learning activities, the effects, student attitude, and the main challenges of K-12 flipped classrooms. In this section, we first compare our findings in K-12 education with the findings in higher education. By synthesizing the practices reported in the reviewed studies, we propose a rudimentary model of flipped classroom approach. We then offer a set of 10 guidelines (Table 7) to address the possible challenges of K-12 flipped classrooms based on the voices of flipped classroom practitioners together with the relevant literature. These guidelines are grouped into three themes proposed by Betihavas et al. (2016): (1) student-related challenges; (2) faculty challenges; and (3) operational challenges.

**Table 7:** Summary of the guidelines of implementing K-12 flipped classrooms

Category	Guidelines
Student-related challenges	1. Opening up teacher-student communication before flipping 2. Demonstrating students how to learn through flipped classroom 3. Using cognitive theory of multimedia learning to guide video production 4. Retaining the workload when flipping a course 5. Providing students with communication platform outside the classroom
Faculty challenges	6. Enriching teachers’ knowledge of flipped classroom approach 7. Preparing flipped learning materials progressively
Operational challenges	8. Supporting the students who are limited by technology resources 9. Using LMS with gamification to monitor and motivate student learning 10. Providing institutional supports of operating flipped classrooms

*LMS* learning management system

#### Comparing the flipped classrooms in K-12 education and higher education

The present review overall suggests that the students in K-12 flipped classrooms would have a better achievement, or at least performed equally as in traditional classrooms. This finding was similar to the conclusion of some previous reviews of flipped classroom research in higher education (e.g., Betihavas et al. 2016; O’Flaherty and Phillips 2015).

Unlike some higher education contexts such as Seery’s (2015) review study on chemistry flipped classrooms, the present review cannot draw “an overwhelming agreement that students liked the approach” (p. 762) in K-12 education. While student attitude toward flipped classroom approach was generally positive, some studies reported that a few students preferred traditional teaching approach because of the inability to ask questions during video lectures and students being accustomed to traditional instruction (Schultz et al. 2014). In particular, DeSantis et al. (2015) found that their students generally reacted negatively toward the change of instructional approach. Meanwhile, the instructional videos produced by their team members “did not feature the host teacher” (p. 51). Student satisfaction in their flipped classroom was thus significantly lower than that in its traditional counterpart.

As for the challenges of implementing flipped classrooms, most of the problems occurred in higher education were found also in the contexts of K-12 education. For the student-related challenges, some K-12 students were unreceptive with the structure of flipped classroom approach as in higher education (Giannakos et al. 2014). Also, students had a negative feeling regarding the amount of out-of-class preparation time as in nursing education (Betihavas et al. 2016). For the video lecture, there is a need for K-12 teachers to design carefully the instructional videos since their students may be disengaged by watching long videos (Kettle 2013; Schultz et al. 2014; Snyder et al. 2014). Also, K-12 students might need to ask questions during video lectures (Bhagat et al. 2016; Schultz et al. 2014). Concerning the group activities inside the classroom, K-12 students might require more guidance on group process in order to work as productive as university students (Grypp and Luebeck 2015). In the present review, no studies reported a decrease of attendance, as stated in Giannakos et al.’s (2014) review, after using flipping a course. However, the regular attendance may be due to the strict regulation of K-12 schools rather than the use of flipped classroom approach.

Faculty challenges in K-12 flipped classrooms were similar to higher education. First, flipped classroom approach requires a high initial cost particularly regarding the production of instructional videos (Betihavas et al. 2016; Giannakos et al. 2014; O’Flaherty and Phillips 2015). Second, teachers should be sufficiently trained in using flipped classroom approach in order to put this approach into full use (Zuber 2016).

When compared with higher education, more operational challenges were identified in the contexts of K-12 education. Similar to the rural and remote university students (Betihavas et al. 2016), a few students in K-12 flipped classrooms also suffered from limited Internet access. Meanwhile, K-12 teachers may have difficulties in monitoring student learning outside the classroom. They may also encounter technical problems and require supports from schools when operating their flipped course.

#### A rudimentary model of flipped classroom approach

**Fig. 3:**
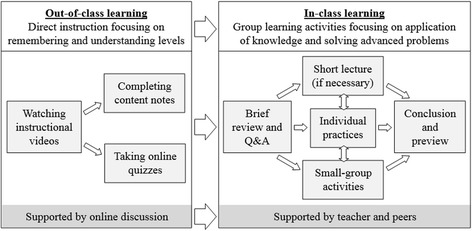
A proposed model of flipped classroom approach

Based on the practices reported in the reviewed studies, we propose a rudimentary model of flipped classroom approach (Fig. 3). In order to be practical in most of the K-12 education contexts, we assume the following: (1) only basic IT resources (e.g., video production, Internet access) are available. Therefore, the flipped classroom model would not draw upon any special functions of some self-developed systems (e.g., Lai and Hwang 2016); (2) the flipped course is taught by only one teacher. Therefore, team teaching practices (e.g., Kirvan et al. 2015) would not be considered.

The central teaching strategy in the out-of-class learning component is direct instruction (Bishop and Verleger 2013; Kirvan et al. 2015) focusing on the knowledge levels of remembering and understanding (Lai and Hwang 2016). Students learn the course materials by watching instructional videos. Teachers can provide content notes to guide students’ note-taking (DeSantis et al. 2015) and ensure students have adequately prepared for class meetings (Clark 2015). Toward the end of out-of-class learning, teachers can provide online exercises for learning evaluation (Wang 2016). By checking students’ online learning performance, teachers can “conduct some discussion based on any misunderstandings or high-error-rate questions in class” (Lai and Hwang 2016, p. 129–130). To support students’ out-of-class learning, teachers can provide students with communication platform for asking questions (guideline 5).

As for the in-class learning component, teachers can first have a brief review on video lecture to recall students’ memory and clarify any misunderstanding (e.g., DeSantis et al. 2015; Grypp and Luebeck 2015; Lai and Hwang 2016). Then, most of the time can be spent on group learning activities (Bishop and Verleger 2013) focusing on applying the knowledge learned from video lectures (Lai and Hwang 2016) and solving advanced problems (Chao et al. 2015; Clark 2015) under the supports of teacher and peers. For example, group discussion (Bhagat et al. 2016; Lai and Hwang 2016) and collaborative tasks (Clarks 2015) can be used inside the classroom. Nevertheless, teachers can still offer hands-on exercises for students’ individual practices (Clark 2015) since solving problem independently is also important for their learning. In some occasions, teachers can consider delivering short lecture to introduce course contents (Tsai et al. 2015) and extend students’ knowledge (Lai and Hwang 2016). For example, Schultz et al.’s (2014) students suggested “difficult concepts be presented in class and not through video” (p. 1339). Perhaps, it is suitable for teachers to explain complicated concepts inside the classroom. In this way, teachers can have immediate understanding on how students grasp the knowledge by observing their facial cues, and further elaborate the difficult parts according to students’ enquiries. Finally, teachers can conclude the class (Huang and Hong 2016) or ask students to gather in groups and review what they have learned (DeSantis et al. 2015). Teachers may also have a brief preview on the out-of-class learning items for the next lesson (Huang and Hong 2016) to promote student interest.

Nevertheless, we suggest incorporating the flipped classroom model with the following guidelines to prevent some potential challenges. For example, we propose using 6-min videos (guideline 3) and limiting the pre-class activities of each lesson within 20 min (guideline 4). These strategies can avoid students’ disengagement from video lecture and overloading students in class preparation.

#### Addressing student-related challenges

##### Guideline 1: Opening up teacher-student communication before flipping

Unlike traditional classroom, flipped classroom approach requires students to explore course content before class. Students thus have more autonomy to schedule their learning and more in-class time for peer interactions together with the teacher’s assistance. However, some students did not understand the rationale of re-ordering the teaching and learning activities (Snyder et al. 2014; Wang 2016). Also, some students were not familiar with the arrangement of a flipped course, which may affect the efficacy of this instructional approach (Clark 2015; Schultz et al. 2014; Snyder et al. 2014).

At the beginning of implementation, teacher-student communication is necessary to promote students’ acceptance of this instructional approach. On one hand, teachers should detail the goal of flipped classroom approach as well as its routines and procedures (Clark 2015; Mazur et al. 2015). For example, Mazur et al. (2015) would provide a detailed overview of course requirements together with an explanation of the steps involved. On the other hand, students should have a chance to express their concerns about the flipped course. In this way, teachers can address students’ worries and provide any necessary help or guidance.

##### Guideline 2: Demonstrating students how to learn through flipped classroom

Clark (2015) reported that it was demanding and challenging for students to pick up a new learning approach and understand course content at the same time. As Grypp and Luebeck (2015) observed in their high school calculus course, “even these academically advanced students needed further instruction on how to work together productively and maximize the benefits of this new learning model” (p. 192). Therefore, it is necessary to first demonstrate how flipped classroom approach works to students.

In Kirvan et al.’s (2015) practice, they would prepare their students gradually before full implementation of their flipped classroom. Students were asked to view a video lecture during class time. At the same time, they introduced students with some cognitive skills such as making their own notes while watching the video lectures. Providing instructor brief notes to accompany the videos is another useful technique to guide student learning during video lectures (Grypp and Luebeck 2015; Kirvan et al. 2015; Snyder et al. 2014). For the in-class activities, students may not be accustomed to the change especially regarding the group learning process (Grypp and Luebeck 2015). Teachers should provide clear instructions to ensure better communication and efficiency in group activities. For example, Clark (2015) would divide his students into three groups according to their ability. Each group of students had a clear lesson objective and completed their corresponding learning tasks. The high ability students worked on practice problems in groups without the teacher’s assistance, whereas the average students first reviewed the contents with the teacher before doing in-class exercises. As for the underperforming students, they would revisit the instructional videos in groups and gain a better understanding of the materials. In this example, every student in Clark’s (2015) flipped classroom knew their own learning objective and what to be discussed with their group members.

##### Guideline 3: using cognitive theory of multimedia learning to guide video production

Some students were disengaged when watching long instructional videos (Kettle 2013; Schultz et al. 2014). Concerning the video presentation, a few students complained to Snyder et al. (2014) that “I feel like I’m just reading and listening to facts, rather than you talking to us in person” (p. 314). In this regard, Mayer’s (2014) cognitive theory of multimedia learning can inform the design of instructional videos in flipped classrooms.

Mayer’s (2014) proposed 12 design principles to enhance the multimedia instructions. For example, segmenting principle stresses that a long presentation should be divided into a series of short videos. Specifically, empirical findings suggested that students’ median engagement time of watching instructional videos was 6 min (Guo et al. 2014). Thus, the desirable length of each video should be within 6 min. Also, personalization principle suggests that the presentation in videos should be spoken in a conversational style. Teachers should use an informal conversation with students (e.g., “I” and “you”), instead of a non-personalized style speaking in a third-person formal monologue. In addition, signaling principle states that learning is enhanced when essential materials are highlighted. Teachers may consider using PowerPoint-embedded presentation such as screencasts (Grypp and Luebeck 2015; Schultz et al. 2014; Snyder et al. 2014). It can offer a step-by-step instruction to guide students’ video watching (Grypp and Luebeck 2015) and assist students in note-taking (Snyder et al. 2014).

##### Guideline 4: retaining the workload when flipping a course

Echoing the findings of previous reviews in higher education (e.g., Betihavas et al. 2016; O’Flaherty and Phillips 2015), some K-12 students were upset that the pre-class workload of flipped classrooms overwhelmed their time at home (Schultz et al. 2014; Snyder et al. 2014; Wang 2016). Teachers should retain, as in its traditional format, the workload of their flipped course.

We encourage teachers to estimate the time required for the homework that traditionally done outside the classroom. Teachers can use this time requirement as a reference when designing their out-of-class learning activities of flipped classrooms. In addition, empirical studies in higher education suggested that the total time of all video segments for each lecture should be confined to about 20 min (McGivney-Burelle and Xue 2013; Vazquez and Chiang 2015). In this way, teachers can ensure that students would not be frustrated because of the extra workload.

##### Guideline 5: providing students with communication platform outside the classroom

Some students lamented that they could not ask questions during pre-class activities (Bhagat et al. 2016; Schultz et al. 2014). Different from traditional classroom, students in a flipped classroom environment cannot interrupt their teacher for enquiries or seek for further elaboration while watching instructional videos. To overcome this problem, teachers can create an online discussion forum for students to post their questions and discuss with peers (Bhagat et al. 2016). The learning community can thus be extended outside the classroom.

#### Addressing faculty challenges

##### Guideline 6: enriching teachers’ knowledge of flipped classroom approach

Among the reviewed studies, some teachers recalled their experiences as a first-time user of flipped classroom approach (e.g., Chen 2016; Clark 2015; Grypp and Luebeck 2015; Kettle 2013). At the initial stage, teachers may neither understand the value of flipped classroom approach nor accustom to this new instructional approach. As Grypp and Luebeck (2015) suggested, teachers “must first embrace the inherent value of this new structure and explore new uses of class time” (p. 192).

Institutes can create opportunities for teachers to share their experiences of implementing flipped classrooms as well as to receive feedback from colleagues or other professionals (Mazur et al. 2015). In Kirvan et al.’s (2015) study, a student teacher joined the teaching team of their flipped classroom. By enacting the flipped course, the student teacher gained experiences in both video production and lesson design. Kirvan et al. (2015) concluded that their intervention could be a critical component of teacher preparation and “may be important for making education theory come alive for new teachers” (p. 219). Therefore, institutes may consider strengthening their teacher training and professional development on flipped classroom approach.

##### Guideline 7: preparing flipped learning materials progressively

In some K-12 flipped courses, preparing flipped learning materials required considerable start-up effort (Chen 2016; Kettle 2013; Kirvan et al. 2015; Snyder et al. 2014). Chen (2016) explained that although there were instructional videos such as Kahn Academy available online, “not all of the topics taught in high school had all of the video resources for flipped classroom” (p. 417). It was also “not an easy task to find videos that perfectly match what a teacher wants his or her students to learn” (p. 418). Consequently, a substantial amount of teacher time was required to create their own materials.

Before flipping the entire course, teachers can start small and proceed at a reasonable pace (Grypp and Luebeck 2015; Snyder et al. 2014). Experiment in small ways also enables teachers to gain experiences of using flipped classroom approach (Grypp and Luebeck 2015). Teachers can cumulate the flipped learning materials by working on two to three topics every year. Grypp and Luebeck (2015) further recommended teachers flipping their courses in team. In other words, teachers can share their experiences of implementing flipped classrooms as well as their teaching resources. However, in DeSantis et al.’s (2015) experience, the materials created by others may not feature the host teacher. Discussion and agreement on the materials designed are thus necessary if teachers intend to develop a flipped course collaboratively.

#### Addressing operational challenges

##### Guideline 8: supporting the students who are limited by technology resources

As some reviewed studies revealed, not all K-12 students have Internet access to view the pre-class videos at home (Chen 2016; Clark 2015; Kettle 2013; Snyder et al. 2014; Wang 2016). Wang (2016) cautioned that “learners with less family support may lose the chance of learning” (p. 412) in flipped classrooms. Teachers should consider students’ socio-economic status and make IT supports available for students. For example, teachers can extend the use of computer facilities in school to support the implementation of flipped classrooms (Schultz et al. 2014). Also, teachers can prepare a few additional copies of flipped learning materials in flash drives or DVDs for the students who do not have Internet connection at home (Clark 2015; Schultz et al. 2014).

##### Guideline 9: using LMS with gamification to monitor and motivate student learning

Chao et al. (2015) pointed out that “it is difficult to ensure that students had truly previewed the video” (p. 524). In this regard, they designed follow-up quizzes on instructional videos to ensure students had previewed the learning materials. A learning management system (LMS) is therefore required to monitor and record the data of student learning. However, there is still a possibility that students complete the quizzes casually without being well prepared from video lectures. So how can we engage students in learning tasks?

Outside the contexts of flipped classroom approach, gamification is recently used in the education field to engage student in learning (Hamari et al. 2014). Hew et al. (2016) found that digital game elements such as points, badges, and leaderboard could produce a positive effect on student motivation and engagement. In a gamified environment, they found that students would be more active online (e.g., contribute more on discussion forum) and engage in more difficult tasks. Some LMSs such as Moodle enable the use of game elements. To motivate student learning, teachers may consider flipping and gamifying their course by using these systems.

##### Guideline 10: providing institutional supports of operating flipped classrooms

Flipped classroom approach relies on the extent of support and investment by schools in IT resources (Huang and Hong 2016; Wang 2016). For example, Chen (2016) alerted that teachers may encounter problems on video production or “run into issues with being unable to upload the videos” (p. 418). Thus, the support from IT staff is essential when implementing a flipped course.

Institutes may consider allocating additional manpower to support the implementation of flipped classrooms. In this way, teachers can develop their flipped learning materials collaboratively (Grypp and Luebeck 2015) and administer the flipped course in team (Kirvan et al. 2015; Mazur et al. 2015). For example, Kirvan et al. (2015) split their students into two groups (re-teaching group and exploration group) by referring to their daily assessment results. One teacher provided remedial help for students who need further understanding of the materials (re-teaching group), while another teacher helped more capable students explore the materials more deeply (exploration group). Once the re-teaching group was ready to proceed, they would join the exploration group to engage in the advanced learning activities.

## Conclusions

This article reviewed the empirical studies of flipped classroom approach in K-12 education. We provided an overview of their flipped learning activities, the findings about the effects of flipped courses on achievement, student attitude toward flipped classroom approach, and the challenges associated with its implementation. Although the flipped classroom approach is not a panacea for all education ills, it seems to promote active learning which requires students to solve problems using what they had learned before class. In the present review, there is no evidence that flipped classroom approach negatively impact student learning in K-12 education. At best, this instructional approach can help students perform significantly overall better than students in traditional classrooms.

Findings regarding student attitude toward flipped classroom approach are mixed. The negative feedback from students highlights the importance of improving this instructional approach. The challenges of using flipped classrooms were categorized into three main themes, namely student-related challenges, faculty challenges, and operational challenges. Based on the empirical findings and relevant literature, a flipped classroom model and a set of 10 guidelines were formulated to address these potential challenges.

However, the findings of the present review were limited to 15 studies of K-12 flipped classrooms. While the number of flipped classroom studies has been increasing (Giannakos et al. 2014), it appears that the research in K-12 education occupies only a small portion of the body of literature. In particular, only two studies of elementary school flipped classrooms (i.e., Lai and Hwang 2016; Tsai et al. 2015) were found in our search. Moreover, we cannot identify any challenges reported in these two studies. More empirical studies are recommended to investigate the effects and challenges of K-12 flipped classrooms, especially in the contexts of elementary school.

The future research should address the major limitations of some previous studies. For example, researchers should utilize a pre-test in their comparison study to evaluate the initial equivalence among groups, instead of merely assuming that the different groups are similar in terms of student prior knowledge. Also, future studies should investigate consecutive uses of flipped classroom approach with a longer time frame (Bhagat et al. 2016; Clark 2015).

One possible research method to examine and evaluate the use of flipped classroom approach over a longer time frame (e.g., 1 year or more) is design-based research (Anderson and Shattuck 2012; Mazur et al. 2015). Design-based research allows a researcher to iteratively adjust and improve a flipped course. This could potentially yield a more in-depth understanding of the effects of the instructional approach on student achievement and attitude as compared to a one-off experiment or quasi-experiment design. Conducting a design-based research over a longer of time could also yield more rigorous practical guidelines for using flipped classroom approach in K-12 settings. In addition to the STEM field, directions for future studies can focus on other subject domains of K-12 education such as language learning (Huang and Hong 2016).

## Appendix 1

Table 8 shows the major findings of the reviewed K-12 flipped classroom studies.

**Table 8:** Major findings of the reviewed K-12 flipped classroom studies

Study	Student achievement	Student attitude
Bhagat et al. (2016)	Students’ achievements in FC were significantly higher than TC. Low achievers in FC preformed significantly better than that in TC.	Students’ motivations in FC were significantly higher than TC.
Chao et al. (2015)	Students’ achievements in FC were significantly higher than TC.	FC students’ learning attitudes, motivation, and self-evaluation were enhanced.
Chen (2016)	No significant difference between FC and TC in test scores.	Students in FC had more discussion and interaction during the class time.
Clark (2015)	No significant difference between FC and TC in academic performance.	Students responded favorably to FC and experienced an increase in their engagement and communication when compared to TC.
DeSantis et al. (2015)	No significant difference in learning outcomes between FC and TC.	TC students reported significantly higher satisfaction with their learning than FC.
Grypp and Luebeck (2015)	Student learning and achievement in FC were at least equivalent to TC.	The depth and equity in group interactions were increased in FC. No overwhelming consensus about which mode of instruction preferred.
Huang and Hong (2016)	FC students’ ICT and English reading comprehension improved significantly.	
Kettle (2013)	Findings about student achievement were mixed.	FC students considered taking notes and working through problems in class as effective and enjoyable, whereas watching videos was the least effective and least enjoyable.
Kirvan et al. (2015)	Learning gains were statistically significant and similar in both FC and TC.	
Lai and Hwang (2016)	Students’ post-test score in SRFC was significantly higher than FC.	Students’ self-efficacy in SRFC was significantly higher than FC.
Mazur et al. (2015)		By emphasizing collaborative learning, group work and accessibility, FC could engage students in inquiry-based learning
Schultz et al. (2014)	A statistically significant difference was found on all assessments with FC performing higher on average than TC.	Most students had a favorable perception about FC.
Snyder et al. (2014)		FC increased student engagement, instruction in career and college technological skills, and facilitation of special education students’ needs.
Tsai et al. (2015)	The effect of FPBL on improving students’ learning performance was significantly higher than TC and PBL.	
Wang (2016)	Students in both FC and MAFC significantly improved their Chinese performance.	Student motivation in MAFC was better than FC in terms of self-directed preview learning.

*FC* flipped classroom, *FPBL* problem-based learning with flipped classroom, *MAFC* mobile-assisted flipped classroom, *PBL* problem-based learning, *SRFC* self-regulated flipped classroom, *TC* traditional classroom

## Appendix 2

Table 9 illustrates the flipped learning activities in the reviewed K-12 flipped classroom studies.

**Table 9:** Flipped learning activities in the reviewed K-12 flipped classroom studies

Study	Pre-class	In-class	After-class
Bhagat et al. (2016)	Watching 15–20-min videos	Group discussion on textbook problems	
Chao et al. (2015)	Watching videos, answering 5–8 online short quizzes	Q&A on videos, group discussion on ill-structured problems, presentation	
Chen (2016)	Watching videos	Practicing skills through textbook activities, journal writing, worksheets	
Clark (2015)	Watching videos, listening to podcasts, reading articles, viewing presentations, completing content notes	Independent practice, group work, hands-on activities, discovery learning, project-based learning, real-world applications	
DeSantis et al. (2015)	Watching videos, completing worksheets with fill-in-the-blank and MC questions	Group discussion on video content, 2Q&A on videos, individual tasks, class discussion	
Grypp and Luebeck (2015)	Watching 6–10-min videos, reading textbook	Brief introduction and review, hands-on activities, group problem solving assignments	
Huang and Hong (2016)	Watching videos	Warm-up discussion, group work, students’ questioning and giving feedback, teacher’s conclusion	
Kettle (2013)	Watching videos, taking notes	Problem solving	
Kirvan et al. (2015)	Watching videos	Pre-assessment, re-teaching for underperforming students, collaborative learning, gallery walks/carousel activities, investigation/inquiry problems	
Lai and Hwang (2016)	Learning and taking quizzes from e-books, watching videos	Clarifying students’ misunderstandings, extending students’ knowledge	Students’ self-evaluation and reflection
Mazur et al. (2015)	Watching videos	Group discussion on problems, presentation	
Schultz et al. (2014)	Watching 10–15-min screencast, online reflection	Brief review, problem solving	
Snyder et al. (2014)	Watching 8–12-min screencast, taking notes	Brief review, group work, presentation, class discussion, inquiry-based learning	
Tsai et al. (2015)	Watching 10-min videos, online discussion	Group discussion on assigned tasks, introducing course contents	
Wang (2016)	Visiting multimedia learning contents, online questions, taking notes	Discussion, sharing learning thought	
